# Research progress on nonsteroidal anti-inflammatory drugs in the treatment of pituitary neuroendocrine tumors

**DOI:** 10.3389/fphar.2024.1407387

**Published:** 2024-07-24

**Authors:** Jiaqi Li, Xinkang Shi, Tao Tang, Manxin Zhou, Feng Ye

**Affiliations:** ^1^ Department of Neurosurgery and Neurocritical Care Medicine, Deyang People’s Hospital, Deyang, China; ^2^ Department of Neurosurgery, YiDu Central Hospital of Weifang, Weifang, China; ^3^ School of Medical and Life Sciences, Chengdu University of Traditional Chinese Medicine, Chengdu, China; ^4^ Clinical Medicine School of Chengdu Medical College, Chengdu, China; ^5^ Sichuan Clinical Research Center for Neurological Diseases, Deyang, China

**Keywords:** Pituitary neuroendocrine tumor, nonsteroidal anti-inflammatory drugs, celecoxib, cyclooxygenase, antitumor

## Abstract

Pituitary neuroendocrine tumor is the third most common primary intracranial tumor. Its main clinical manifestations include abnormal hormone secretion symptoms, symptoms caused by tumor compression of the surrounding pituitary tissue, pituitary stroke, and other anterior pituitary dysfunction. Its pathogenesis is yet to be fully understood. Surgical treatment is still the main treatment. Despite complete resection, 10%–20% of tumors may recur. While dopamine agonists are effective in over 90% of prolactinomas, prolonged use and individual variations can lead to increased drug resistance and a gradual decline in efficacy, which ultimately requires surgical intervention. Nonsteroidal anti-inflammatory drugs reduce the production of inflammatory mediator prostaglandins by inhibiting the activity of cyclooxygenase and exert antipyretic, analgesic, antiplatelet, and anti-inflammatory effects. In recent years, many in-depth studies have confirmed the potential of nonsteroidal anti-inflammatory drugs as a preventive and antitumor agent. It has been extensively utilized in the prevention and treatment of various types of cancer. However, their specific mechanisms of action still need to be fully elucidated. This article summarizes recent research progress on the expression of cyclooxygenase in pituitary neuroendocrine tumors and the treatment of nonsteroidal anti-inflammatory drugs. It provides a feasible theoretical basis for further research on pituitary neuroendocrine tumors and explores potential therapeutic targets.

## Background

A pituitary neuroendocrine tumor (PitNET) originates from anterior pituitary cells. With the popularization of autopsy and routine radiological examination, its incidence rate is increasing, accounting for approximately 15% of primary intracranial tumors. Its main clinical manifestations include abnormal hormone secretion symptoms, symptoms caused by tumor compression of the surrounding pituitary tissue, pituitary stroke, and other anterior pituitary dysfunction. Surgery, medication, radiotherapy, and chemotherapy are the primary therapeutic approaches to treat PitNETs. Dopamine receptor agonists, such as bromocriptine and cabergoline, are commonly used as first-line drugs for the treatment of prolactinomas, a type of PitNET. While most patients with prolactinomas experience shrinkage of the pituitary tumor and normalization of prolactin levels after taking these medications, approximately 15% of prolactinoma patients will develop drug resistance, which ultimately leads to the need for surgical intervention (Vroonen et al., 2012). Thus, it is of great significance to study the molecular mechanisms and signal transduction pathways of PitNET progression.

Nonsteroidal anti-inflammatory drugs (NSAIDs) constitute a class of anti-inflammatory medications that do not contain steroid structures. NSAIDs can be categorized into non-selective COX inhibitors (e.g., aspirin, ibuprofen, and meloxicam) and selective COX-2 inhibitors (e.g., celecoxib and nimesulide). These medications exhibit various effects, such as antipyretic, analgesic, anti-platelet aggregation, anti-rheumatic, anti-inflammatory, and antitumor properties (Grosser et al., 2017). Their primary mechanism of action is to inhibit the activity of cyclooxygenase (COX) and prevent the breakdown of arachidonic acid (AA) into prostaglandins (PGs) and thromboxane. PGs mediate physiological and pathological processes, such as tumor development, neovascularization promotion, and inflammation through their interaction with the E protein receptor (EP) and the subsequent modulation of cAMP and protein kinase production (Onguru et al., 2004). On the other hand, thromboxane has the ability to elicit platelet aggregation, stimulate cell proliferation, and induce vasoconstriction (Onguru et al., 2004). COX, a member of the myeloperoxidase family, comprises two subtypes: COX-1 and COX-2. COX-1 is widely expressed in mammalian tissues and is primarily involved in the synthesis of prostaglandins and vital physiological functions; In contrast, COX-2 is typically expressed in central nervous systems, including neurons, glial cells, and cerebral blood vessels. However, its expression is significantly upregulated in response to pathological stimuli, such as inflammation and tumors (Bazan, 2001). Ongoing research on COX-2 has revealed its role in promoting tumor cell proliferation, inhibiting cell apoptosis, promoting tumor neovascularization, dedifferentiation, and enhancing tumor invasion and metastasis (Liu et al., 2015). An international consensus statement stated that regular use of conventional NSAIDs can reduce cancer incidence rates (Cuzick et al., 2009). With mounting evidence, it is becoming increasingly apparent that the overexpression of COX-2 plays a pivotal role in tumorigenesis and tumor progression, indicating that targeting COX-2 inhibition may serve as a potential strategy to mitigate the risk of cancer development (Aliabadi et al., 2023; Xu et al., 2024). Studies have demonstrated that overexpression of COX-2 is a prevalent occurrence in diverse malignancies, such as colorectal cancer (Nanda and Dhawan, 2021), breast cancer (Liu et al., 2019), bladder cancer, head and neck tumors, melanoma, and other malignant tumors (Liu et al., 2015; Nanda and Dhawan, 2021). However, the carcinogenic mechanisms of COX-2 and the antitumor mechanisms of COX-2 inhibitors have not been fully elucidated. These mechanisms can be broadly categorized into COX-dependent and non-COX-dependent signaling pathways. Currently, there is a dearth of research exploring the effects of NSAIDs on PitNET, and conclusive findings are yet to be established. Although there are some clues from basic research, the precise antitumor mechanism of NSAIDs still requires further exploration. This article provides a comprehensive review of the latest research advancements on NSAIDs in PitNET. It discusses the expression of COX in pituitary tumors, and investigates the potential mechanisms of action of COX-2 inhibitors, offering fresh perspectives and directions for subsequent research.

## COX is highly expressed in PitNET

Clinical studies have demonstrated that COX-1 and COX-2 are significantly overexpressed in PitNET tissue, respectively exhibiting a 1.78-fold and 4.4-fold increase, compared to normal pituitary tissue. The expression levels of these enzymes are closely associated with age and tumor type (Akbari et al., 2020). Notably, there is no research exploring the link between COX-1 and tumorigenesis and progression. Interestingly, COX-1 expression is elevated in invasive pituitary tumors, but no significant differences have been observed across different pituitary tumor types, microadenomas, and macroadenomas (Akbari et al., 2020). Research has also revealed a positive correlation between COX-2 expression and patient age. Specifically, COX-2 expression is significantly higher in PitNET patients aged 50 years and above than those younger than 50. This age difference is particularly pronounced in non-functional pituitary tumors, but there is no statistically significant difference in functional pituitary adenomas (Zhao et al., 2020). The COX-2 expression in non-functional pituitary adenomas is significantly higher than that in functional pituitary adenomas; The expression level of COX-2 in adrenocorticotropic hormone adenoma is weaker than that in prolactinoma and growth hormone adenomas, both of which are higher than normal pituitary adenomas (Onguru et al., 2004; Akbari et al., 2020). Additionally, COX-2 expression is higher in large pituitary adenomas compared to microadenomas, with statistically significant differences (Akbari et al., 2020). However, there is no statistically significant difference in COX-2 expression based on patient gender, tumor sizes, or Knosp grades (Vidal et al., 2003; Zhao et al., 2020). COX-2 staining is more intense in multi-hormone pituitary adenomas compared to mono-hormone pituitary adenomas (Bloomer et al., 2003). COX-2 expression is displayed in the cytoplasm of cells in dual immunofluorescence co-localization (Zhao et al., 2020). Similar to the high expression of COX-2 in various malignant tumors, PitNET also exhibits overexpression of COX-2. It is hypothesized that COX-2 overexpression may be a contributing factor to the development of pituitary adenomas, although it does not seem to have a significant impact on their progression or invasiveness. and provides a theoretical basis for the application of NSAIDs in the treatment of pituitary adenomas.

## NSAIDs exert antitumor effect by inhibiting COX activity and reducing prostaglandin production

In recent years, COX-2 and prostaglandin E_2_ have played vital roles in the formation of tumor tissue. In chronic inflammation, COX breaks down arachidonic acid into various produce prostaglandins (PGs). Prostacyclin (PGI_2_) derived from COX-1 protects the surface of the gastrointestinal mucosa and promotes platelet aggregation leading to thrombus formation. On the other hand, prostaglandin E2 (PGE_2_) derived from COX-2 serves as a mediator in both acute and chronic inflammation and is closely associated with various malignancies (Wang and Dubois, 2006). Increasing research among various prostaglandins has found that PGE_2_ exerts its biological functions by binding to the EP receptor, a type of G protein-coupled receptor, and mediating its downstream signaling pathway (Wang and Dubois, 2006). Specifically, it can induce tumor cell proliferation, suppress apoptosis, promote angiogenesis, and suppress immune responses. Furthermore, PGE_2_ activates the Ras-MAPK signaling pathway by binding to EP receptors. The Ras-MAPK signaling pathway holds a critical position in the eukaryotic signal transduction network, exerting a crucial regulatory influence on cell proliferation (Wang and Dubois, 2006). NSAIDs have the ability to inhibit COX activity, thereby diminishing the activation of the Ras-MAPK signaling pathway induced by PGE2, effectively suppressing cell proliferation (Husain et al., 2001). Additionally, PGE_2_ can induce the expression of anti-apoptotic protein Bcl-2 and NF-κB protein, which is closely related to tumorigenesis to inhibit apoptosis (Masferrer et al., 2000). The overexpression of COX-2 and the production of PGE_2_ can upregulate the expression of vascular endothelial growth factor (VEGF) and activate the epidermal growth factor receptor (EGFR) to promote neovascularization (Mann et al., 2001; Li et al., 2021). This process provides the necessary oxygen and nutritional support for tumor growth. NSAIDs can mediate the synthesis of PGE_2_ and its downstream related factors by inhibiting COX activity, thereby inhibiting tumor cells through the regulation of multiple signaling pathways. It is worth noting that high expressions of COX-1 and COX-2 have also been found in pituitary tumor cells, providing a potential theoretical basis for NSAIDs to inhibit pituitary tumor cells. However, relevant research has not yet been conducted, and more experimental and clinical data are needed to validate these findings.

## COX-2 involvement in immune evasion

COX-2 plays a significant role in tumor immune evasion and resistance to immunotherapy. This key enzyme promotes the synthesis of PGE_2_ and its binding to EP receptors, subsequently activating a series of membrane receptors to achieve specific cellular biological functions (Liu et al., 2015). COX-2/PGE_2_ significantly inhibits the maturation of dendritic cells and the expression level of MHC-II molecules, suppressing the antigen-presenting ability of dendritic cells and the specific immune response of T cells, thus contributing to the immune evasion of tumor cells (Harizi et al., 2003). In addition, PGE_2_ can also inhibit the cytotoxic killing effect and interferon-γ secretion of NK cells, as well as the anti-tumor activity of macrophages, regulating immune function (Wang and Dubois, 2006; Martinet et al., 2010). Studies have shown that in pituitary tumors, COX-2 may enhance tumor cell immune evasion and exacerbate the invasiveness and malignancy of PitNET by upregulating the expression of programmed cell death protein (Zhao et al., 2020). The impact of NSAIDs on the immune response of PitNET remains an incompletely elucidated area, and further in-depth research is needed to understand the specific mechanisms of action.

## NSAIDs suppress tumors via a COX-independent signaling pathway

Current research indicates that the antitumor effects of NSAIDs may not be limited to the inhibition of COX-2. Instead, these drugs may exert significant antitumor effects through non-COX-dependent signaling pathways at high concentrations, although the specific mechanism has not been fully elucidated (Tegeder et al., 2001). Selective COX-2 inhibitors and non-selective COX inhibitors may differ in their antitumor mechanisms, providing a new direction for exploring their mechanisms of action. NF-κB, as a central mediator of immune responses, plays a crucial role in cancer progression and anticancer treatment. It can regulate the differentiation and survival of immune cells, control gene transcription levels, and regulate apoptotic factors, thereby inducing cell apoptosis (Tegeder et al., 2001). In the study of colorectal cancer, celecoxib can directly activate NF-κB and its dependent gene transcription without relying on COX inhibition, activate the Fas/FasL signaling pathway to mediate cell killing, and also inhibit protein kinase B phosphorylation and promote cell apoptosis (Han et al., 2000). In contrast, the mechanism of aspirin is different. It inhibits IKKβ phosphorylation and subsequently suppresses NF-κB activity (Tegeder et al., 2001). Aspirin activates the p38-MAPK pathway in colorectal cancer cells, leading to rapid ubiquitination and degradation of cyclin D1, which in turn activates the NF-κB pathway to mediate cell apoptosis (Thoms et al., 2007). This difference may be related to the specificity of cell types. Additionally, aspirin mediates the formation of the antiapoptotic protein Bcl-2 and FKB-2 complex through non-COX-dependent signaling pathways, induces the nuclear translocation and phosphorylation of Bcl-2, and mediates the overexpression of the pro-apoptotic protein Bax gene, thereby promoting the apoptosis of tumor cells (Choi et al., 2013). In colon cancer research, selective COX-2 inhibitors can downregulate the expression of cell cycle proteins and upregulate the expression of cell cycle inhibitory proteins p21waf1 and p27kipl, inhibiting the transition of colon cells from the G0/G1 phase to the S phase in a concentration-dependent manner (Grösch et al., 2001). This antitumor effect is independent of COX-2 expression, further demonstrating the diversity of NSAIDs’ antitumor mechanisms. Although there have been studies on the antitumor effects of celecoxib and aspirin in other malignancies, the mechanisms are diverse and complex. However, research on the inhibitory effects of NSAIDs on PitNET and the related mechanisms is still limited. Therefore, further exploration and research in this field are needed to provide theoretical support and practical guidance for the development of more effective antitumor drugs in the future.

## Methylation of COX-2 in PitNET

In recent years, epigenetic mechanisms have become increasingly important, especially in the occurrence and development of cancer. The main epigenetic mechanisms include DNA methylation, histone modification, and functional noncoding RNA. Among them, the DNA methylation process refers to the transfer of methyl groups from adenosylmethionine to cytosine bases under the catalytic action of DNA methyltransferase (DNMT). This methylation reaction is reversible and provides cells with the ability to adjust gene expression at any time when needed. On the contrary, the DNA demethylation process refers to the oxidative demethylation of cytosine catalyzed by tet methylcytosine dioxygenase (TET) (Ito et al., 2011). There are many unstable intermediates in this catalytic process, among which 5-hydroxymethylcytosine (5hmC) is a relatively stable intermediate. Therefore, 5hmC is a biomarker indicating DNA demethylation, and the ratio of 5hmC to 5 mC indicates the demethylation status (Ito et al., 2011). In the regulation of DNA methylation, the UHRF protein family plays an important role. UHRF1 can cooperate with DNMT to regulate the DNA methylation process, while UHRF2 serves as an auxiliary factor of TET to participate in the regulation of DNA demethylation (Polepalli et al., 2019). These proteins are crucial in maintaining the dynamic balance between DNA methylation and demethylation. DNA methylation is vital in various biological processes, including the regulation of gene expression, cell differentiation, and development, as well as tumorigenesis and tumor progression (Szabó et al., 2020). More and more scholars are beginning to discover the close relationship between PitNET and DNA methylation. Through comprehensive genome-wide quantitative analysis, Duong and team (2012) found a large number of abnormal methylation sites in pituitary adenomas, which are mainly concentrated in regions rich in cytosine and guanosine phosphate in the genome (CpG islands). The degree of methylation within CpG islands is much higher than at sites outside CpG islands (Duong et al., 2012). There is also a significant difference in DNA methylation levels between nonfunctional pituitary adenomas and functional pituitary adenomas, which provides new clues for us to understand the pathogenesis of pituitary adenomas (Tost et al., 2014). In addition, regardless of the histological subtype of pituitary adenomas, their proliferation level is closely related to DNA methylation levels. In pituitary adenomas with high proliferation and low differentiation, we observed an increase in the expression of UHRF1 and DNMT proteins, accompanied by a decrease in the ratio of 5hmC/5 mC (Szabó et al., 2020). This further confirms the important role of DNA methylation in the occurrence and development of PitNET. There was no significant difference in overall DNA methylation levels between invasive and non-invasive pituitary adenomas, and no statistically significant difference was observed in the Knosp grading of pituitary adenomas. This indicates that the overall DNA methylation profile cannot independently predict the invasiveness of PitNET (Szabó et al., 2020). With the deepening of research on epigenetic mechanisms, it appears that aspirin can indirectly promote an elevation in the expression of the TET enzyme and its cofactor UHRF2 through a COX-independent pathway, and reduce the mRNA and protein expression levels of pituitary tumor transforming gene (PTTG), thereby increasing the level of 5hmC and increasing the overall degree of DNA demethylation (Szabó et al., 2022). Consequently, aspirin exerts antitumor effects through these mechanisms. In summary, epigenetic mechanisms play an important role in the occurrence and development of PitNET, and NSAIDs are gradually being discovered for DNA methylation, bringing new ideas and strategies for the treatment of PitNET.

## NSAID inhibits tumor proliferation through miRNA regulation

MicroRNAs (miRNAs) are a class of single-stranded noncoding small RNAs containing 20–25 nucleotides that regulate gene expression and protein synthesis at the post-transcriptional level by binding to 3′-UTPs of target mRNA (Saliminejad et al., 2019). They play a pivotal role in various tumor-related processes, including tumor inhibition or proliferation, drug resistance, metastasis, cell apoptosis, and signal transduction. Additionally, they can contribute significantly to diagnosis, treatment, and prognosis evaluation. In a systematic review and meta-analysis, research has demonstrated that COX-2 plays a role in promoting tumorigenesis and development by regulating miRNA and its downstream pathways. Additionally, NSAIDs can also suppress tumor growth by regulating the upregulation or downregulation of miRNA (Mishan et al., 2020). In endometrial cancer, Liu Ying and her colleagues (2018) demonstrated that miR-101 negatively regulates COX-2 protein expression, and COX-2 inhibitors can reverse the inhibitory effect of miR-101 on tumor growth (Liu et al., 2018). It can be seen that there is a certain correlation between the interaction between miRNA and COX-2 in the occurrence and development of tumors. Experimental research data show that celecoxib, a selective COX-2 inhibitor, can inhibit tumor proliferation, migration, invasion, and epithelial-mesenchymal transformation through miR-145/TGFBR2/Smad3 signaling pathway in bladder cancer cells (Liu et al., 2019). Meanwhile, it elucidated that the tumor suppressor factor miR-145-5p makes prolactinoma sensitive to bromocriptine by downregulating tumor protein translationally controlled (Jian et al., 2019). Currently, it remains unclear whether NSAIDs can inhibit tumor growth by regulating the expression of miRNA-145 in PitNET, and further experiments are needed to verify this hypothesis. Although reports have analyzed the differential expression of miRNAs between PitNET and normal pituitary tissue samples using microarray and real-time PCR techniques, and explored the potential functions of these differential miRNAs through bioinformatics methods (Bottoni et al., 2006), there is still a lack of high-throughput sequencing research data on miRNAs after NSAIDs treatment for PitNET.

## Diagnosis and treatment prospects

To evaluate the diagnostic value of COX-1 and COX-2 in PitNET, Nasrin Akbari et al. (2020) used the ROC curve to show that COX-2 and PGE2 levels can significantly distinguish pituitary tumors from normal tissues (Akbari et al., 2020). The effectiveness of combining COX-2 with other tumor markers for diagnosing PitNET requires further research on a large population to evaluate its accuracy and specificity in early detection of PitNET. The evaluation of the effectiveness of NSAIDs in treating PitNET requires more experimental data and evidence-based medicine.

## Conclusion and future directions

The treatment methods for PitNET include surgical treatment, drug therapy, and radiation therapy. Currently, about 25% of patients undergoing surgical treatment may have residual tumors and recurrence; 10%–15% of patients develop resistance to medication. Therefore, it is particularly important to choose a drug with significant therapeutic effects and minimal adverse reactions. NSAIDs are commonly used antipyretic and analgesic drugs, which are widely used in clinical practice to treat various diseases such as fever, pain, rheumatoid arthritis, and osteoarthritis. Many epidemiological studies have shown that NSAIDs have both anti-tumor and preventive effects, and their mechanisms of action can be divided into two categories: one is dependent on the COX signaling pathway, and the other is independent of the COX-2 signaling pathway (Liggett et al., 2014). There is no relevant epidemiological research data on the anti-tumor therapeutic effect of NSAIDs on PitNET. PitNET tissue has been identified to have high expression levels of COX-1 and COX-2, theoretically providing potential targets for NSAIDs. To delve deeper into the precise role of NSAIDs in PitNET, future research undoubtedly requires a series of rigorous and systematic *in vitro*, *in vivo*, and clinical studies for validation. Specifically, the blood-brain barrier permeability of celecoxib, and its ability to effectively cross this crucial physiological barrier, holds significant implications for its therapeutic potential in treating PitNET. This review provides an in-depth exploration of the expression status of cyclooxygenase in PitNET and the research progress in nonsteroidal anti-inflammatory drug therapy. Key highlights include the capacity of these drugs to inhibit tumor cell proliferation, induce tumor cell apoptosis, impede tumor neovascularization, modulate tumor immunity, regulate miRNA expression, and even effectuate demethylation. While NSAIDs’ antitumor mechanisms have been thoroughly explored in malignancies such as colorectal cancer, lung cancer, and breast cancer, their potential applications in treating pituitary neuroendocrine tumors and other benign neoplasms still require further exploration. Overall, research on the impact of NSAIDs on PitNET is still in its early stages, and further research is needed to investigate the molecular mechanisms and signal transduction pathways of NSAIDs in PitNET progression. By understanding the potential biology of these tumors, we aim to identify new therapeutic targets and develop more effective drugs to provide more effective treatment options for patients with pituitary tumors, thereby improving their quality of life.
